# *Sinorhizobium meliloti* BR-bodies promote fitness during host colonization

**DOI:** 10.1128/mbio.02490-25

**Published:** 2025-10-31

**Authors:** Kaveendya S. Mallikaarachchi, Jason L. Huang, Shanmukha Madras, Rodrigo A. Cuellar, Zhenzhong Huang, Alisa Gega, Imalka W. Rathnayaka-Mudiyanselage, Vidhyadhar Nandana, Nadra Al-Husini, Natalie Saldaña-Rivera, Loi H. Ma, Eric Ng, Klara Christensen, Nima Pendar, Sophia Li, Nayeli Rodas Deleon, Joseph C. Chen, Jared M. Schrader

**Affiliations:** 1 Department of Biology, Indiana University 1772, Bloomington, Indiana, USA; 2 Departments of Chemistry and Biological Sciences, Wayne State University 2954, Detroit, Michigan, USA; 3 Department of Biology, San Francisco State University 7147, San Francisco, California, USA; University of California Berkeley, Berkeley, California, USA

**Keywords:** BR-bodies, microbe-host interaction, mRNA decay rate, *Sinorhizobium meliloti*, nitrogen fixation, liquid-liquid phase separation, degradosome

## Abstract

Biomolecular condensates are non-membrane-bound assemblies of proteins and nucleic acids that facilitate specific cellular processes. Like eukaryotic P-bodies, the recently discovered bacterial ribonucleoprotein bodies (BR-bodies) organize the mRNA decay machinery in α-proteobacteria; however, the similarities in molecular and cellular functions across species have been poorly explored. Here, we examine the functions of BR-bodies in the nitrogen-fixing endosymbiont *Sinorhizobium meliloti*, which colonizes the roots of compatible legume plants. Similar to *Caulobacter crescentus*, assembly of BR-bodies into visible foci in *S. meliloti* cells requires the C-terminal intrinsically disordered region (IDR) of RNase E *in vivo* and *in vitro*, and foci fusion is readily observed *in vivo*, suggesting that they are liquid-like condensates that form via mRNA sequestration. Using Rif-seq to measure mRNA lifetimes, we found a global slowdown in mRNA decay in a mutant deficient in BR-bodies, indicating that compartmentalization of the degradation machinery promotes efficient mRNA turnover across α-proteobacteria. Although BR-bodies are constitutively present during exponential growth, the abundance of BR-bodies increases upon cell stress, whereby they promote resistance to environmental stresses. Finally, we show that BR-bodies enhance competitive fitness during *Medicago truncatula* root colonization and appear to be required for effective symbiosis, as mutants without BR-bodies failed to promote robust plant growth on nitrogen-free medium. These results suggest that BR-bodies provide a fitness advantage for bacteria during host colonization, perhaps by enabling better resistance against the host immune response.

**IMPORTANCE:**

Although eukaryotes often organize their biochemical pathways in membrane-bound organelles, bacteria generally lack such subcellular structures. Instead, membraneless compartments called biomolecular condensates have recently been found in bacteria to organize and enhance biochemical activities. Bacterial ribonucleoprotein bodies (BR-bodies), as one of the most characterized bacterial biomolecular condensates identified to date, assemble the mRNA decay machinery via the intrinsically disordered regions (IDRs) of proteins. However, the implications of such assemblies are unclear. Using a plant-associated symbiont, we show that the absence of BR-bodies results in slower mRNA decay, sensitivity to environmental stresses, and ineffective symbiosis, suggesting that BR-bodies play critical roles in regulating biochemical pathways and promoting fitness during host colonization.

## INTRODUCTION

Eukaryotic and bacterial cells have been found to compartmentalize their mRNA decay machinery into biomolecular condensates (1–3), non-membrane-bound organelles that organize critical cellular processes, including RNA degradation and storage. Bacterial ribonucleoprotein bodies (BR-bodies) were the first biomolecular condensate discovered in bacteria, composed of the RNA degradosome complex (4). The large intrinsically disordered region (IDR) of RNase E was found to be necessary and sufficient for BR-body formation, and bioinformatic analyses of other bacteria suggest that BR-bodies are likely widespread across phylogeny (2, 4–7), including other RNases like RNase Y and J (8, 9). Despite the observed presence of intrinsically disordered regions across RNA degradosome scaffolds, the sequences are not conserved (2), and RNA degradosome clients vary dramatically between species (2), making it unclear whether BR-bodies maintain the same functions across species. Initial characterization of BR-bodies was performed in *Caulobacter crescentus*, where it was shown that they assemble with poorly translated mRNAs and promote the rapid decay of mRNA (10, 11). Although constitutively present, BR-bodies were also stimulated to assemble in various stress conditions, where they promoted resistance (4). Although BR-bodies have been identified in other bacteria, including *Sinorhizobium meliloti* (4), the functional role of BR-bodies in mRNA decay and stress resistance has not been thoroughly tested outside of *C. crescentus*. Interestingly, transposon insertions or targeted mutations in RNase E that truncate the large IDR and likely abolish BR-body assembly have been found in diverse species to cause stress sensitivity, defects in host colonization, and other pleiotropic phenotypes (4, 12–18). This suggests that BR-bodies may promote similar molecular and cellular phenotypes across species.

*S. meliloti* is an α-proteobacterium that can live freely or in symbiosis with legume plants (19, 20). In order to colonize compatible hosts, *S. meliloti* must invade plant root tissue and survive host defenses, such as antimicrobial peptides (21–24). Following reciprocal signaling events between the bacterium and plant, *S. meliloti* establishes chronic infection by inducing the formation of root nodules and colonizing them (19). After engulfment by plant cells in the nodules, changes in gene expression reprogram the bacteria for differentiation into nitrogen-fixing bacteroids, allowing the mutualistic infection to promote robust plant growth (25). In addition to transcriptional regulation of nitrogen fixation genes, post-transcriptional regulation by small RNAs has been found to impact a variety of cellular processes, including metabolism, the cell cycle, and quorum sensing (26, 27). RNase E in *S. meliloti* has been found to affect quorum sensing and S-adenosylmethionine homeostasis (28, 29). Although the protein appears to be essential for viability, its C-terminal IDR can be removed with insertion of a plasmid or mini-Tn5 at the 3′ end of the *rne* gene (13, 28). Microarray analysis of wild type and a mini-Tn5 insertion mutant identified only a small subset of mRNAs with altered steady-state levels when the IDR of RNase E was disrupted (29). However, measurements of steady-state mRNA cannot distinguish differences in the rates of mRNA turnover. Moreover, it has not been established whether the *S. meliloti* RNase E IDR is necessary for BR-body formation *in vivo* or whether BR-bodies impact plant colonization and symbiosis.

To examine the conservation of molecular and cellular phenotypes affected by BR-bodies, we generated a C-terminal IDR truncation mutation in *S. meliloti* RNase E. We found that the IDR was necessary for BR-body formation and that the IDR deletion mutant showed a general slowdown in mRNA decay, suggesting that BR-bodies promote mRNA turnover. Through a combination of drug and stress treatments, we determined that *S. meliloti* BR-bodies are constitutively present, stimulated by poorly translated mRNA, and more strongly induced under a variety of stresses. Altogether, these results are in line with prior work in *C. crescentus*, suggesting that BR-bodies promote similar functions across species despite their IDRs only sharing 28.86% sequence identity. Finally, we found that BR-bodies promote fitness during stress and *Medicago truncatula* root colonization. Altogether, these results suggest that many of the molecular and cellular activities facilitated by BR-bodies are likely conserved across bacterial species, enhancing viability across diverse lifestyles.

## RESULTS

### *S. meliloti* RNase E forms BR-bodies that promote mRNA decay

**Fig 1:**
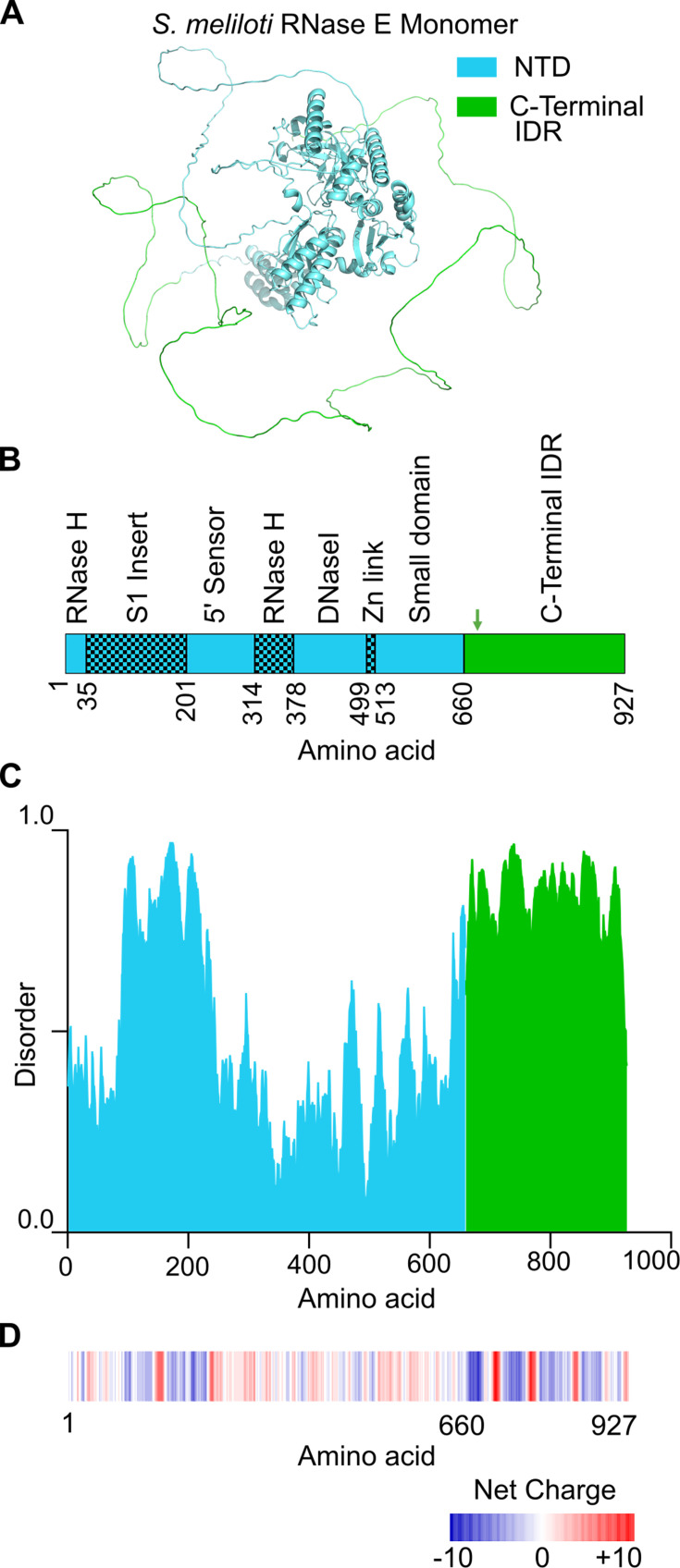
*S. meliloti* RNase E has the features necessary for phase separation and BR-body formation. (**A**) RNase E monomer structure predicted from AlphaFold (32, 33). The folded catalytic nuclease domain is colored in cyan, whereas the IDR region is highlighted in green. (**B**) RNase E domain structure. Domain annotations were from Hardwick et al. (30). Briefly, the N-terminal catalytic region of RNase E contains two RNase H domains, an S1 domain insert, a 5′ sensor, a DNase I domain, a zinc link, and a small domain (30). Green arrow indicates amino acid 665, where the protein was truncated in the RNase EΔIDR mutant. (**C**) Disorder across RNase E. dSCOPE predictions of the RNase E disorder (31). (**D**) Charge patterning across RNase E. The IDR is highly enriched with alternating charge patches, which were shown to be necessary and sufficient for phase separation in *C. crescentus* RNase E (4).

**Fig 2:**
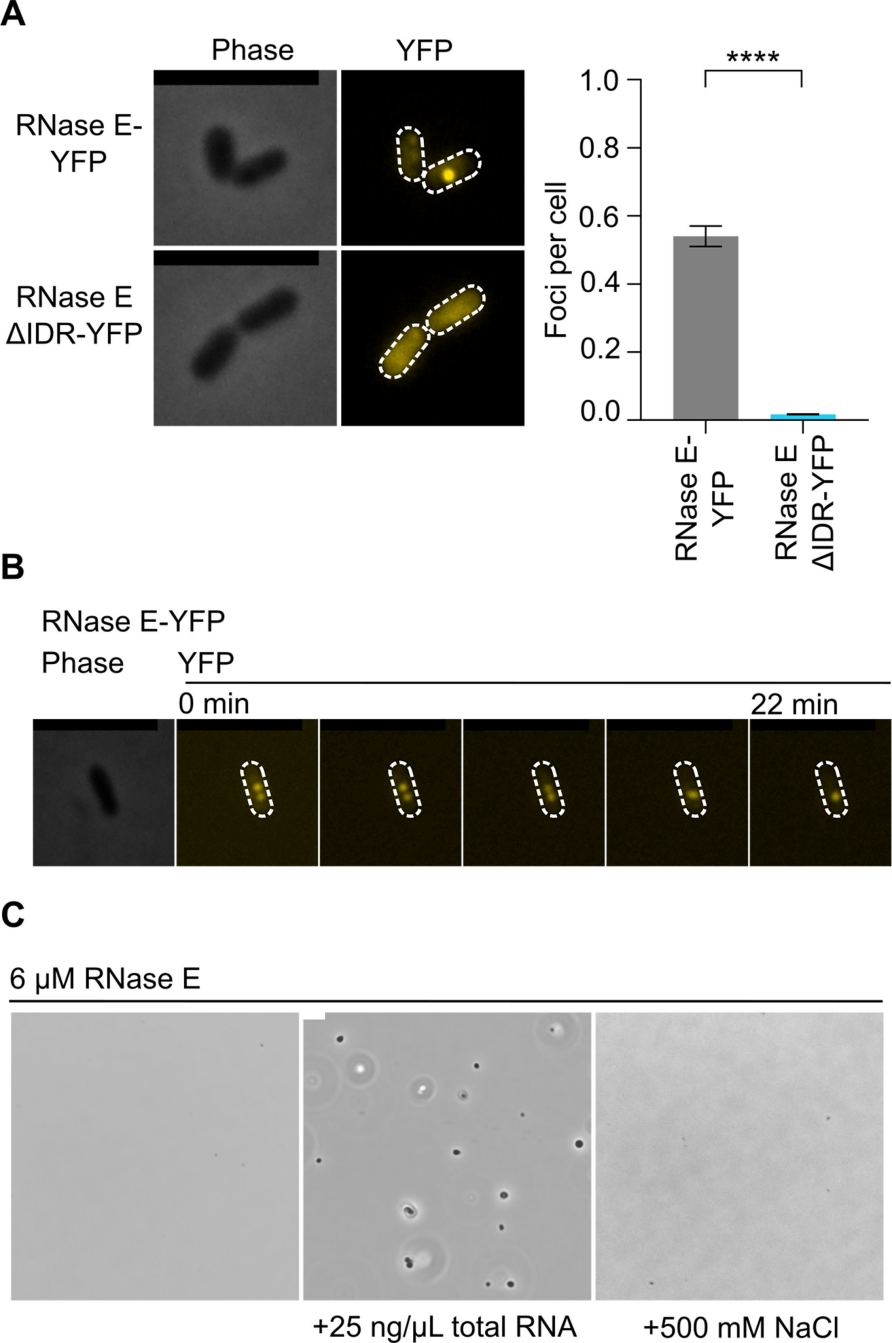
*S. meliloti* RNase E requires the C-terminal IDR to assemble into dynamic BR-bodies. (**A**) RNase E foci require the C-terminal IDR. Rm1021 cells harboring YFP fusion to the C-terminus of either full-length RNase E or RNase EΔIDR were examined by phase contrast and fluorescence (YFP) microscopy. Foci quantitation was performed using MicrobeJ, 326 cells were used for RNase E-YFP, and 295 cells were used for RNase EΔIDR-YFP. p-values were calculated with a *t*-test (****: *P* < 0.0001, two-tailed, unequal variance). Error bars represent standard errors. The black scale bars in the micrographs indicate 5 µm. (**B**) Representative fusion of two RNase E-YFP foci suggests phase separation. Two RNase E-YFP foci were imaged using time-lapse microscopy. Droplets were observed to fuse together and relax into a spherical droplet over 22 min. More than 5 fusion events were observed during this time. Scale bar is 5 µm. (**C**) *In vitro* phase separation of *S. meliloti* RNase E. Purified RNase E undergoes *in vitro* phase separation in the presence of RNA, and these RNase E condensates readily dissolve upon the addition of 500 mM NaCl. Images were captured via phase contrast microscopy. The white scale bar on the phase contrast image is 5 µm.

α-proteobacterial RNase E proteins contain an N-terminal catalytic domain and a large IDR in the C-terminal domain, which is necessary and sufficient for phase separation into BR-bodies in *C. crescentus* (Fig. 1A and B) (2, 4). To generate a mutant of *S. meliloti* that cannot assemble BR-bodies, we truncated the IDR at codon 665 based upon the domain annotation (30) and predicted region of disorder from dSCOPE (31) (Fig. 1B and C). This IDR has been defined previously to contain an alternating pattern of “charge patches” of positively and negatively charged amino acids, which promote RNase E phase separation through electrostatic interactions (Fig. 1D) (2, 4). The IDR deletion mutant (RNase EΔIDR) was viable and showed no detectable difference in growth rate compared with wild type in TY medium (Fig. S1A and B). To examine the importance of the IDR in BR-body formation *in vivo*, the localization of RNase E-YFP and RNase EΔIDR-YFP was determined by fluorescence microscopy. RNase E-YFP showed robust formation of fluorescent foci, whereas RNase EΔIDR-YFP led to a near total loss of foci compared with the wild type (Fig. 2A). Consistent with previous observations in the related α-proteobacterium *C. crescentus* (4), this result suggests that the RNase EΔIDR mutation abolishes the ability to form BR-bodies *in vivo*. A western blot with anti-GFP antibody confirms that both RNase E-YFP and RNase EΔIDR-YFP retain the YFP tag and are expressed at comparable levels, indicating that the diffuse fluorescence of the ΔIDR variant reflects a failure to assemble BR-bodies, not tag cleavage or reduced expression (Fig. S1C). *C. crescentus* RNase E foci fusion events were observed via time-lapse microscopy, suggesting the foci are phase-separated condensates *in vivo* (4). To test if *S. meliloti* RNase E can phase separate *in vivo*, we performed time-lapse microscopy with the RNase E-YFP strain (Fig. 2B). Here, we similarly observed that *S. meliloti* RNase E-YFP foci could fuse like those of *C. crescentus,* where two foci with 2D areas of 0.12 µm² and 0.079 µm² increased to 0.26 µm² post-fusion, suggesting that they also form phase-separated condensates *in vivo*. Although focus fusion alone is not sufficient evidence of phase separation, *C. crescentus* RNase E was also shown to phase separate with RNA *in vitro* (4, 11). To test the intrinsic capacity for *S. meliloti* RNase E to phase separate, we purified *S. meliloti* full-length RNase E and RNase EΔIDR and confirmed that they were both active using a 5S rRNA processing assay (Fig. S2A). We then assayed the proteins, along with the catalytically inactive RNase E IDR domain, for condensate formation with RNA and observed that the IDR is both necessary and sufficient for phase separation *in vitro* (Fig. 2C; Fig. S2B). A combination of genetic mutations and drug treatments suggested that *C. crescentus* RNase E competes with ribosomes *in vivo* for access to mRNAs, which act as the major substrate for BR-body formation (4, 10). To test the impact of mRNA availability in *S. meliloti*, we performed growth assays in different conditions, including drug treatments with rifampicin, which blocks transcription and rapidly depletes the cell of mRNA, and chloramphenicol, which freezes ribosomes on mRNAs and traps mRNAs in polysomes (4). Upon short treatment of rifampicin (100 µg/mL for 30 min), we saw a strong reduction in the number of BR-bodies (Fig. S3), as we previously reported (4); treatment with chloramphenicol (200 µg/mL for 30 min) yielded similar results (Fig. S3). These observations suggest that *S. meliloti* BR-bodies assemble via the accumulation of poorly translated mRNAs with RNase E, as in *C. crescentus* (4).

**Fig 3:**
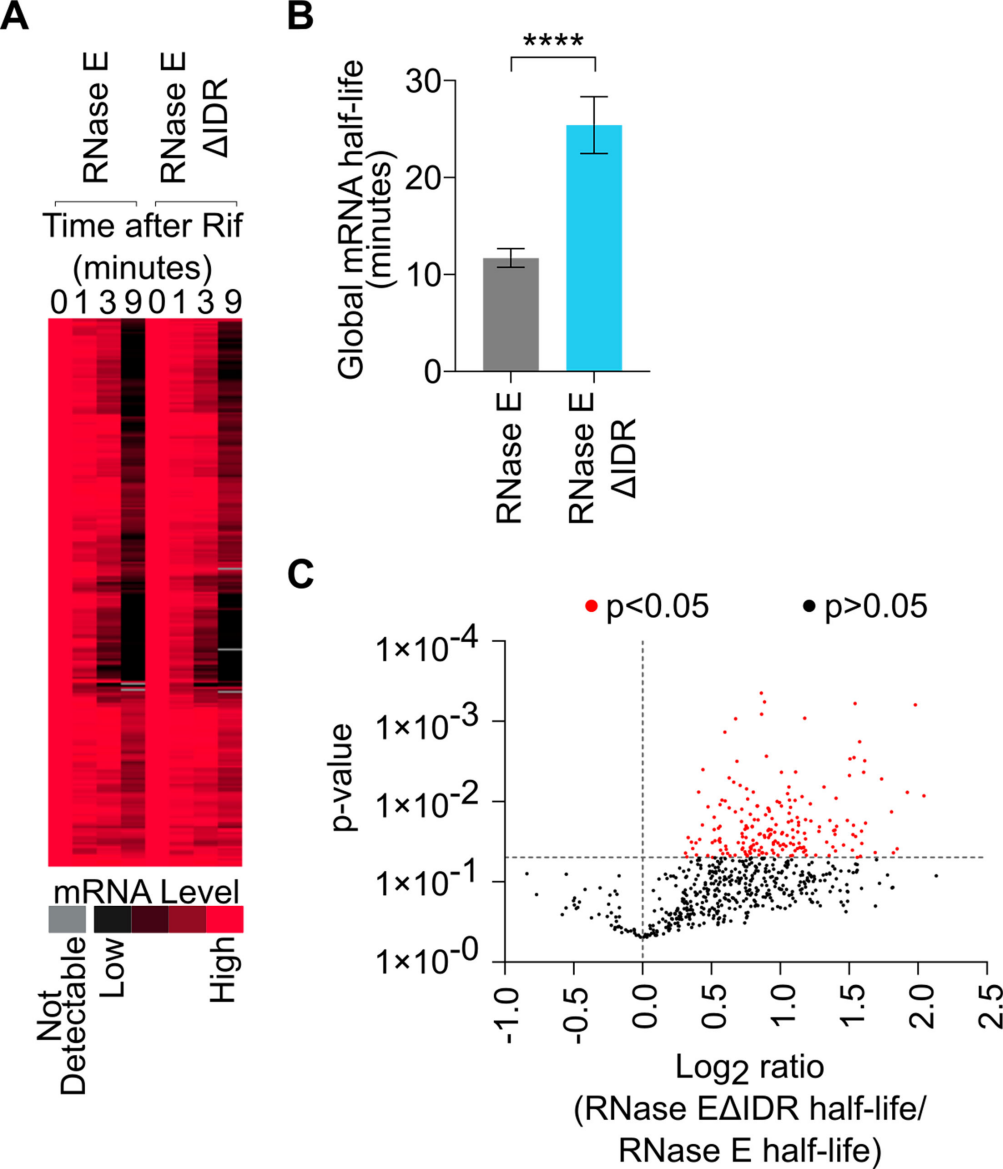
The BR-body-deficient mutant (RNase EΔIDR) shows a global slowdown in mRNA decay. (**A**) Rif-seq using RNase E and RNase EΔIDR strains. RNA was extracted from cells incubated with rifampicin (Rif) at the indicated time points. RNA abundance was normalized to the level in the cells prior to rifampicin treatment and displayed as a heat map by k-means clustering. Each horizontal line represents a single mRNA. (**B**) The BR-body-deficient mutant (RNase EΔIDR) exhibits a significantly higher global mRNA half-life compared with wild type (RNase E). The global mRNA half-life was calculated from a summation of mRNA reads divided by total RNA reads (mRNA read counts + tRNA read counts) across the time points. Half-lives were estimated by curve fitting, and the average half-life between both biological replicates is presented. Error bars indicate standard deviations, and the p-value was calculated using a two-tailed *t*-test with unequal variance. (****: *P* < 0.0001) (**C**) Fold changes of mRNA half-lives show broad stabilization in the mutant. The half-lives of 665 mRNAs could be measured in both the RNase E and RNase EΔIDR strains; 199 had significantly longer half-lives in the RNase EΔIDR compared with RNase E (*P* < 0.05, based on one-tailed *t*-test with unequal variance). Another 422 out of 665 also exhibited longer half-lives in the RNase EΔIDR, but they were not significantly different from the corresponding values in the wild type (*P* > 0.05). The remaining 44 mRNAs showed shorter half-lives in the mutant compared with RNase E wild type, but none were statistically significant.

BR-bodies facilitate rapid mRNA turnover by compartmentalizing the RNA degradosome together with its substrate mRNAs, increasing their local concentrations (2, 4, 10). Consistent with a role in stimulating mRNA decay, RNase EΔIDR mutants have been shown to have slow mRNA decay in different bacterial species, including *E. coli* and *C. crescentus* (10, 34). To test whether the *S. meliloti* BR-body-deficient mutant (RNase EΔIDR) also exhibits a slowdown in mRNA decay, we performed Rif-seq experiments to measure global mRNA half-lives (10, 35) (Fig. 3A and B). In this assay, wild-type (termed RNase E henceforth) and RNase EΔIDR cells were incubated with rifampicin to block transcription, and RNA abundance was measured prior to and 1, 3, and 9 min after the addition of rifampicin. The half-lives of RNAs can then be calculated in a genome-wide manner (10, 35). Importantly, each time course was collected in duplicate, and tRNA is not removed from the RNA samples, as this stable RNA acts as a loading control. The bulk mRNA half-lives can be calculated from the mRNA fraction, or all the summed mRNA reads divided by total reads (mRNA + tRNA), at each time point. The mRNA fraction of the samples across the time course is then fit to a half-life equation to calculate the bulk mRNA half-lives. The bulk mRNA half-lives show that mRNA is significantly stabilized in the RNase EΔIDR mutant (from half-life of 11.7 min in RNase E to 25.4 min in the RNase EΔIDR mutant) (Fig. 3B). By using the Rifcorrect software package (35), we calculated the half-lives of individual mRNAs expressed in mid-log cells (Fig. 3C). Rifcorrect yielded a total of 665 mRNA half-lives that were measured in all replicates of both the RNase E and RNase EΔIDR Rif-seq data sets (Fig. 3C). Of these 665 mRNAs with calculated half-lives in the RNase E and RNase EΔIDR mutant, 199/665 mRNAs were significantly more stable in the RNase EΔIDR mutant compared with RNase E (*P* < 0.05, one-tailed *t*-test, unequal variance), whereas none of the mRNA half-lives were significantly destabilized in the RNase EΔIDR mutant. As a representative example, the Rif-seq data for the *dnaA* mRNA show a rapid 4.9 min half-life in the wild-type strain and a longer half-life in the RNase EΔIDR mutant (15.8 min) (Fig. S4A). The 199 mRNAs stabilized in the RNase EΔIDR mutant background were subjected to functional GO-term enrichment analysis by NCBI DAVID (36), where we observed two significant categories, protein biosynthesis (*P* = 3.5 × 10^−3^) and pyrimidine biosynthesis (*P* = 5.0 × 10^−2^). In *C. crescentus*, BR-body enriched RNAs were identified to be longer, poorly translated mRNAs, which were found to be stabilized when the IDR was deleted (10). Similarly, in *S. meliloti*, we examined the stabilized mRNAs for their lengths and translational efficiency. We observed that the stabilized mRNAs were significantly longer than mRNAs not stabilized (Fig. S4B, left), as judged by a one-tailed *t*-test of unequal variance (*P* < 0.0001). Using ribosome profiling measurements (37), we also observed less efficient translation of mRNAs that were stabilized in the absence of the RNase E IDR (Fig. S4B, right) (*P* < 0.01, one-tailed *t*-test, unequal variance), despite differences in strain backgrounds and growth conditions. (Ribosome profiling was performed with *S. meliloti* strain 2011 grown semi-aerobically in GMS minimal medium, whereas Rif-seq was performed with Rm1021 grown in TY rich medium.) Taken altogether, the BR-body-deficient mutant (RNase EΔIDR) shows a significant global slowdown in mRNA decay, predominantly of longer, poorly translated mRNAs, suggesting that *S. meliloti* BR-bodies also promote faster mRNA degradation of such mRNAs.

### The BR-body-deficient mutant (RNase EΔIDR) is sensitive to stress and defective in host colonization

**Fig 4:**
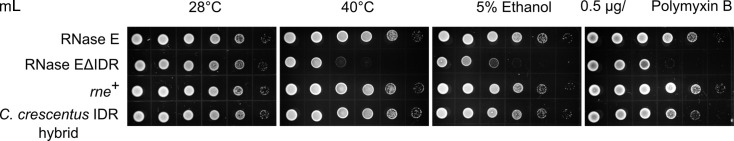
The BR-body-deficient mutant (RNase EΔIDR) is sensitive to multiple stresses. Cells were grown overnight in TY, diluted to an OD600 of 0.5, and 10-fold serially diluted. 5 µL of each dilution was spotted on plain TY plates or TY plates containing 5% ethanol or 0.5 µg/mL polymyxin B and incubated at 28°C or 40°C. The BR-body-deficient (RNase EΔIDR) mutant is more sensitive to various stresses compared with the wild-type (RNase E), *rne*^+^ (derivative of RNase EΔIDR in which the IDR deletion was repaired using the *S. meliloti* RNase E IDR at the *rne* locus), and *C. crescentus* IDR hybrid (derivative of RNase EΔIDR in which the IDR deletion was repaired using the *C. crescentus* RNase E IDR) strains. Black and white images of the plates are used in the figure for clarity.

*C. crescentus* BR-bodies were found to be present during normal growth but more strongly induced under different cellular stresses, promoting a fitness advantage in those conditions (4). To test the effects of stress on *S. meliloti*, we first assessed whether *S. meliloti* BR-bodies were induced under similar conditions. As in *C. crescentus*, we observed an increase in *S. meliloti* BR-bodies with various stresses tested (Fig. S5). The induction of BR-bodies under various stresses suggests that they may also play a role in *S. meliloti* fitness. Therefore, we compared the growth of wild type (RNase E) and the BR-body-deficient mutant (RNase EΔIDR) under various stresses (Fig. 4). To ensure that observed phenotypes were due to the deletion of the RNase E IDR, and not a secondary mutation, we repaired the IDR in the BR-body-deficient mutant (RNase EΔIDR) background, generating the *rne*^+^ strain. In addition, to distinguish the phase separation and RNA degradosome scaffolding activities of the IDR, we replaced the IDR in the BR-body-deficient mutant with the *C. crescentus* RNase E IDR, generating the *C. crescentus* IDR hybrid strain. Because the IDR shares low sequence homology between the two species (28.86% identity), the *C. crescentus* IDR should be less competent at binding *S. meliloti* RNA degradosome clients while still retaining the ability to drive phase separation (Fig. S6). Under permissive conditions at 28°C, we did not observe any significant differences in viability or colony size on solid media (Fig. 4) or growth rates in liquid media (Fig. S1A and B). Upon exposing the cells to a variety of stresses, we observed with the RNase EΔIDR mutant a reduction in colony size when grown at higher temperature (40°C), and a strong reduction in CFUs when grown in the presence of ethanol (Fig. 4). In both cases, the *rne*^+^ and *C. crescentus* IDR hybrid strains grew the same as the RNase E wild type, indicating that the IDR deletion causes a reduction in stress tolerance. This suggests that *S. meliloti* BR-bodies provide a fitness advantage during stress and that the stress tolerance phenotype observed in *C. crescentus* is likely conserved across bacteria.

**Fig 5:**
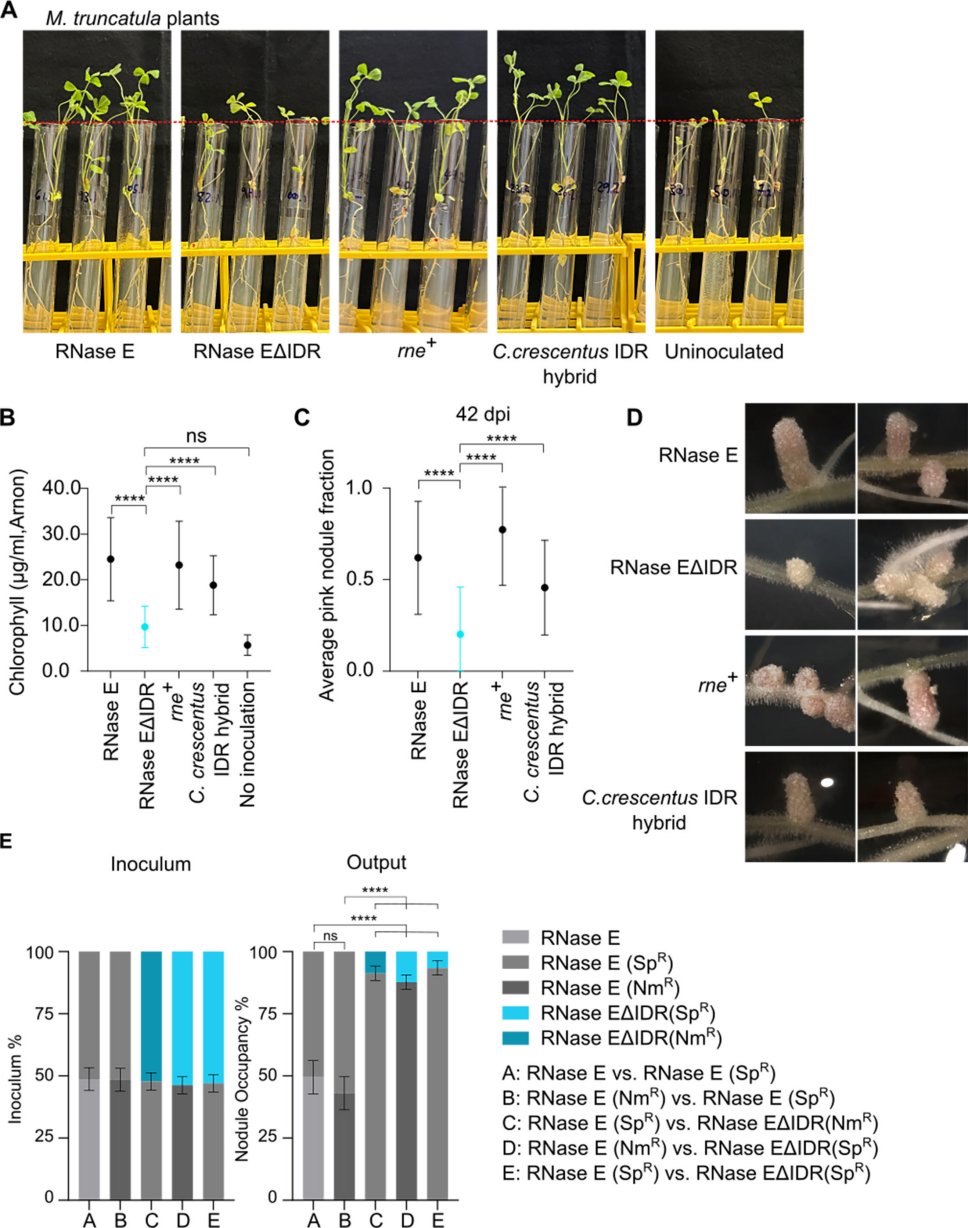
The BR-body-deficient mutant (RNase EΔIDR) is defective in *M. truncatula* root colonization and symbiosis. (**A**) *M. truncatula* plants photographed after being inoculated with the indicated strain of *S. meliloti* and grown on nitrogen-free media for 42 days. Seedlings inoculated with the BR-body-deficient (RNase EΔIDR) strain exhibit less robust growth compared with plants that were inoculated with other strains that can make BR-bodies. The width of each test tube is 18 mm. (**B**) Chlorophyll contents of the plants 42 days post-inoculation. Plants that were inoculated with the RNase EΔIDR strain contain significantly less chlorophyll compared to plants inoculated with the other strains. Arnon’s equations were used to calculate chlorophyll concentrations. Tukey’s multiple comparisons test was used to calculate the *P* values. (****: *P* < 0.0001) (**C**) Fraction of pink nodules per plant inoculated with each strain 42 days post-inoculation (dpi). Seedlings inoculated with strains expressing wild-type RNase E or *C. crescentus* RNase E IDR hybrid had significantly higher fractions of pink nodules compared with seedlings inoculated with the RNase EΔIDR strain (****: *P* < 0.0001, Kolmogorov-Smirnov test). (**D**) Root nodules on *M. truncatula* plants photographed after being inoculated with the indicated strain of *S. meliloti* and grown on nitrogen-free media for 42 days. Uninoculated plants did not develop any nodules. The width of each image is 4 mm. (**E**) The RNase EΔIDR strain is less competitive when colonizing plant root nodules. Competitive symbiosis assays were used to compare the relative colonization efficiency of wild type (RNase E) and the BR-body-deficient mutant (RNase EΔIDR), measured 28 days after seedlings were inoculated with equal mixtures of two strains. The left plot indicates the ratios of the two strains in the inoculating mixtures. The right plot shows mean percentages (± standard deviations) of root nodules colonized by each bacterial strain in distinct competitions. Strains used in the competition were unmarked or marked with resistance to spectinomycin (Sp^R^) or neomycin (Nm^R^). Ratios in the plots were derived from colony counts when the inoculating mixtures or nodule extracts were plated or from colony PCR amplification of the SMc01336 (*rne*) locus. Relative nodule occupancy when RNase E competed against RNase EΔIDR was significantly different than that when RNase E competed against RNase E (ns: not significant, ****: *P* < 0.0001, two-proportion Z-test). A total of three competition replicates were used for wild type vs. RNase EΔIDR (*n* = 318 nodules) and seven competition replicates for wild type vs. wild type (*n* = 587 nodules).

Although *C. crescentus* is a free-living bacterium, *S. meliloti* is a facultative endosymbiont that can invade root tissue and colonize nodules, fixing nitrogen for the host plant to promote growth. To achieve effective symbiosis, *S. meliloti* must adapt to and survive host defenses. The model host *M. truncatula* is known to produce several cationic nodule-specific cysteine-rich (NCR) peptides as a part of its innate immune response against bacteria (21–24). We used polymyxin B, a widely available cationic peptide antibiotic, to mimic these NCR peptides and found that the BR-body-deficient mutant (RNase EΔIDR) was sensitive to polymyxin (Fig. 4), suggesting that BR-bodies may promote a fitness advantage during host colonization. To study the role of BR-bodies during infection, *M. truncatula* seedlings were inoculated with wild type (RNase E), BR-body-deficient mutant (RNase EΔIDR), *rne*^+^, or *C. crescentus* IDR hybrid *S. meliloti* cells, and the plants were grown and monitored on nitrogen-free medium for 42 days (Fig. 5). Seedlings inoculated with the BR-body-deficient mutant (RNase EΔIDR) grew poorly compared with those inoculated with wild type, suggesting that the mutant is unable to form effective symbiosis (Fig. 5A and B). The poor plant growth observed with the RNase EΔIDR mutant was fully alleviated with the *rne^+^* strain, demonstrating that the symbiosis defect was caused by the removal of RNase E’s IDR (Fig. 5A). In addition, the *C. crescentus* IDR hybrid strain also rescued symbiosis, suggesting that RNase E’s ability to phase-separate is likely required for symbiosis, and not the scaffolding of the RNA degradosome (Fig. 5A). As a proxy for plant health, we also measured their chlorophyll contents (Fig. 5B): the RNase EΔIDR mutant led to lower chlorophyll levels, similar to that in plants grown without any *S. meliloti,* and the defect was rescued with either the *rne*^+^ or the *C. crescentus* IDR hybrid strain. Furthermore, we observed similar numbers of root nodules induced in plants inoculated with different strains (Fig. S7), with slightly lower but statistically significant numbers of nodules in *rne^+^*. Plants inoculated with the BR-body-deficient mutant (RNase EΔIDR) exhibited a higher proportion of white nodules instead of healthy pink nodules, suggesting a failure in leghemoglobin production and nitrogen fixation (Fig. 5C and D); again, this defect in the development of pink nodules was rescued by both *rne*^+^ and the *C. crescentus* IDR hybrid (Fig. 5C and D). To examine the symbiosis defect more closely, we performed competitive colonization experiments to compare the relative fitness of the wild-type and RNase EΔIDR strains. We generated 1:1 mixtures of wild type and the BR-body-deficient mutant (RNase EΔIDR), inoculated plants with the mixtures, and recovered bacteria from individual nodules 28 days later (Fig. 5E). Each strain carried a spectinomycin or neomycin resistance marker for rapid identification by plating on selective media. As a control, we also competed unmarked, spectinomycin-marked (Sp^R^) or neomycin-marked (Nm^R^) strains against each other to ensure that the resistance markers did not impact competitive fitness (Fig. 5E). Regardless of the marker, we found that the BR-body-deficient mutant (RNase EΔIDR) showed a large reduction in root nodule occupancy compared with the RNase E strain. In contrast, equal ratios of unmarked, Nm^R^, and Sp^R^ strains were recovered when wild-type RNase E strains competed against each other (Fig. 5E), suggesting that the drug resistance markers did not lead to significant differences in fitness during symbiosis. Thus, the reduction in nodule occupancy by the BR-body-deficient mutant (RNase EΔIDR) compared with wild type reinforces that BR-bodies promote fitness during host colonization (Fig. 5E).

## DISCUSSION

### BR-body cellular functions are conserved in α-proteobacteria

Although RNase E’s IDR was found to be necessary and sufficient for BR-body assembly in *C. crescentus*, and RNase E proteins contain IDRs across bacteria (2), the IDR’s importance in BR-body assembly has not been directly tested in other species. In *S. meliloti*, we showed that the C-terminal IDR is necessary for BR-body assembly *in vivo* and *in vitro* (Fig. 2), allowing our RNase EΔIDR mutant to act as a BR-body-deficient mutant (Fig. 2). In addition, BR-bodies in *S. meliloti* appear to be dynamic, liquid-like condensates, as we observed events of droplet fusion (Fig. 2B), and RNase E droplets are readily dissolved by NaCl *in vitro* (Fig. 2C) or upon treatment with chloramphenicol or rifampicin *in vivo* (Fig. S3). In *C. crescentus*, BR-bodies promote the degradation of long, poorly translated mRNAs (4, 10), and we found that *S. meliloti* BR-bodies similarly accelerate mRNA decay rates of longer, poorly translated mRNAs (Fig. 3; Fig. S4B). BR-bodies are constitutively present during exponential growth; however, they are also stimulated in the presence of cell stresses, whereby they promote stress resistance (Fig. 4; Fig. S5) (4). This suggests that despite the diverse sequences and lengths of IDRs across species (2), the molecular and cellular functions of BR-bodies in mRNA decay appear to be similar across species.

### BR-bodies promote plant colonization and symbiosis

**Fig 6:**
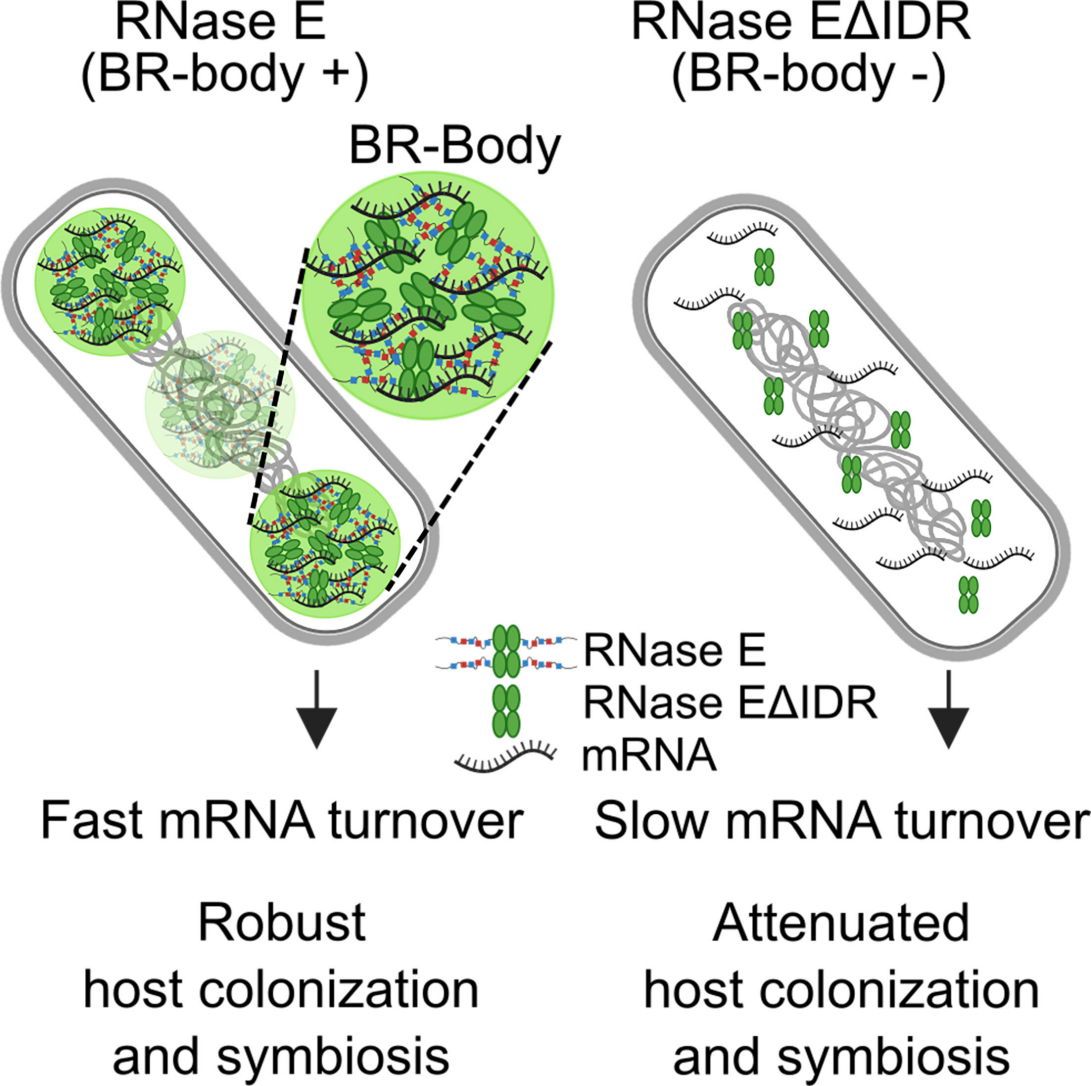
BR-bodies stimulate mRNA decay and promote host colonization. A summary of molecular and cellular phenotypes impacted by the BR-body deficiency, as depicted in both wild type and the RNase EΔIDR mutant. Efficient RNA processing and turnover due to RNase E’s ability to form biomolecular condensates appears critical for *S. meliloti* to colonize host plants and establish effective symbiosis.

Although RNase E mutants with ΔIDR or Tn-insertions that truncate the IDR have been found to have reduced fitness during host colonization in multiple species (12, 14–18), there has not been a direct demonstration that these mutations prevented BR-body formation. Therefore, this study provides the first evidence that BR-body phase separation can promote fitness during host infection (Fig. 6). Although the *S. meliloti* BR-body-deficient RNase EΔIDR mutant can colonize *M. truncatula* roots, it was readily outcompeted by the RNase E wild-type in a direct competition assay, suggesting that there is a significant loss in fitness during symbiosis (Fig. 5). Although the RNase EΔIDR mutant appears to have similar growth to wild type (RNase E) in unstressed conditions *in vitro*, we hypothesize that the stress sensitivity of the mutant makes the bacterial cells more susceptible to the plant’s innate immune defenses, such as antimicrobial peptides (24). This inability to thrive in the host environment manifests as a defect in nitrogen fixation, as the BR-body-deficient mutant failed to stimulate the development of pink nodules and promote plant growth (Fig. 5). Notably, replacing the IDR with that of *C. crescentus* rescued the RNase EΔIDR mutant’s defects in stress resistance and symbiosis. As *C. crescentus*’s IDR is able to support condensate formation but unlikely to serve as a scaffold for the *S. meliloti* RNA degradosome, BR-body phase separation appears critical during host colonization (Fig. 4 and 5). Previous studies demonstrated that sRNAs and their chaperone Hfq play important roles in *S. meliloti* during symbiosis by performing post-transcriptional regulation on key mRNAs (26, 27, 38). BR-bodies may be important organizers of sRNA homeostasis, as sRNAs and Hfq were found to be enriched in *C. crescentus* BR-bodies (10, 11). In addition, RNase E’s IDR is required for regulating multiple *E. coli* sRNAs (39, 40). Interestingly, a similar RNase E IDR truncation mutant in *Brucella abortus* showed attenuated mouse infection and altered sRNA levels (12), suggesting that BR-bodies may also coordinate sRNA activities in animal pathogens.

## MATERIALS AND METHODS

### *S. meliloti* cell growth

All *S. meliloti* strains used in this study (Table 1) were derived from Rm1021 (wild-type RNase E) and were grown at 28°C in Luria-Bertani broth (LB), peptone-yeast extract (PYE), or tryptone-yeast extract (TY) medium, supplemented with gentamicin (Gent), neomycin (Nm), spectinomycin (Sp), and/or streptomycin (Strep) when appropriate (41). All plasmids used in this study are listed in Table 2. Optical density (OD) of liquid cultures was measured at 600 nm in a cuvette using a Nanodrop 2000C or NanoDrop One^C^ spectrophotometer. Strains were analyzed at the mid-exponential phase of growth (OD of 0.3–0.6).

**TABLE 1:** *S. meliloti* strains used in this study

Strain	Relevant genetic markers, features, and/or description	Construction, source, or reference^*a*^
Rm1021	SU47 derivative, Sm^R^ (progenitor of strains listed below)	(42)
JS9	*rne*::pRNE(*Sm*)-YFPC-4 (*rne-yfp*)	(4)
JS377	*rne*::pJSE2 [*rne*(NTD)*-yfp*]	pJSE2 mated into Rm1021
JOE2612	*podJ2*(SMc02231)..Ω (Sp^R^ wild-type strain)	(43)
JOE6312	*podJ2*(SMc02231)..*nptII* (Nm^R^ wild-type strain)	Allelic replacement in Rm1021 using pJC739
JOE6516	*rne*ΔCTD	Allelic replacement in Rm1021 using pJSE3
JOE6526	*podJ2*(SMc02231)..*nptII rne*ΔCTD	Allelic replacement in JOE6312 using pJSE3
JOE6550	*podJ2*(SMc02231)..Ω *rne*ΔCTD	JOE6516 × Φ(JOE2612), select for Sp^R^
JOE6825	*podJ2*(SMc02231)..*nptII rne**^+^***	Allelic replacement in JOE6526 using pJSE4
JOE6826	*podJ2*(SMc02231)..Ω *rne**^+^***	Allelic replacement in JOE6550 using pJSE4
JOE6831	*rne**^+^***	Allelic replacement in JOE6516 using pJSE4
JOE7534	*C. crescentus rne* IDR hybrid	Allelic replacement in JOE6516 using pKM40

^*a*^ Φ indicates generalized transduction, as mediated by bacteriophage ΦN3. For example, JOE6516 × Φ(JOE2612) means that a bacteriophage lysate made from JOE2612 was used to infect JOE6516.

**TABLE 2:** Plasmids used in this study

Plasmid	Relevant genetic markers, features, and/or description	Construction, source, or reference
pJQ200sk	Counter-selectable vector for allelic replacement, *sacB*, Gm^R^	(44)
pVYFPC-4	Integrating plasmid with multiple cloning sites under control of the vanillate promoter, Gm^R^	(45)
pRNE(Sm)-YFPC-4	Integrating plasmid carrying the last 1,101 bp of *rne* from *S. meliloti* Rm1021 in frame with a C-terminal YFP tag, derived from pYFPC-4	(4)
pJC739	pJQ200sk-*podJ2..nptII*, for inserting *nptII* (Nm/Km resistance marker) after *podJ2* gene	This study
pJSE2	Integrating plasmid carrying the last 512 bp of *rne* NTD from *S. meliloti* Rm1021, for fusing the N-terminal domain of RNase E in frame with a C-terminal YFP tag, derived from pYFPC-4	This study
pJSE3	pJQ200sk-*rne*ΔCTD, for allelic replacement to delete the C-terminal domain (IDR) of RNase E	This study
pJSE4	pJQ200sk-*rne*^+^, for allelic replacement to repair the mutation in *rne*	This study
pKM40	pJQ200sk-*C. crescentus rne* IDR, for allelic replacement to make a hybrid IDR strain	This study
Addgene plasmid # 29656	pET His6 MBP TEV LIC cloning vector (1M), used to generate pKM3, pKM44 and pKM45.	http://n2t.net/addgene:29656
pKM3	pET His6 MBP-rne, for purifying full length RNase E	This study
pKM44	pET His6 MBP-rneΔIDR, for purifying RNase EΔIDR	This study
pKM45	pET His6 MBP-rne IDR, for purifying RNase E IDR	This study

For doubling time calculations, Rm1021 derivatives were grown as 25-mL cultures in TY media. Culture tubes were incubated in a shaker incubator at 240 rpm at 28°C. Absorbance at 600 nm was measured every 1 h using a NanoDrop One^C^ spectrophotometer for 14 h. Averages and standard deviations were calculated from readings of three biological replicates of each genotype (RNase E, RNase EΔIDR, *rne*^+^, and *C. crescentus* IDR hybrid).

For acute drug treatment, the log-phase cells were treated with rifampicin (100 µg/mL) or chloramphenicol (200 µg/mL) for 30 min. For serial dilution assays, including cell stress assays, cells were grown overnight in TY media and diluted to an OD of 0.5, followed by 10-fold serial dilutions; 5 µL each of select dilutions was spotted on plates with the indicated composition and incubated at 28 or 40°C. Imaging was done using the Bio-Rad ChemiDoc MP imaging system, and black and white images were used in Fig. 4 for clarity.

### Strain and plasmid construction

#### JS9: Rm1021 *rne::rne-yfp* (Gent^R^)

This strain was generated in a previous study (4).

#### JS377: Rm1021 *rne::(rne(NTD)-*yfp (Gent^R^)

*S. meliloti* RNase E NTD-YFP fusion (RNase EΔIDR-YFP) was generated by amplifying the last 500 bp encoding the RNase E N-terminal domain (NTD) using J5_00418_(SME-NTD)_forward and J5_00419_(SME-NTD)_reverse primers (Table 3), designed using the j5 DNA assembly design automation software (46). The resulting PCR product and pYFPC-4 plasmid (45) were both cut with NdeI and EcoRI, ligated, and transformed into *E. coli* DH5α (Invitrogen). Gent^R^ colonies were then screened by PCR for the NTD insert, and the purified plasmid was verified by Sanger sequencing (Genewiz). This pJSE2 plasmid was then mated into Rm1021 by triparental mating. Desired transconjugants were selected on Gent/Strep plates and verified by PCR and YFP imaging.

**TABLE 3:** Primers used in this study

Name	Sequence (5′−3′)
J5_00418_(SME-NTD)_forward	CGTCCAATTGCATATGCTGATCGTCATCGACTTCATCGACATGG
J5_00419_(SME-NTD)_reverse	CGTAACGTTCGAATTCGCCTCTTCTTCGTCTTCCGGCTCCGG
j5_00743_(CTD_upstream)_forward	TCCCTCGATAAGCTTGATATCGAATTCCTGCAGCGAGATCAAGCGCGACTTCG
j5_00743_(CTD_upstream)_reverse	CGGAGCCGGAAGACGAAGAAGAGTAAGAAGAATAGGAAAAAGCCCG
j5_00743_(CTD_downstream)_forward	CCGGAAGACGAAGAAGAGTAAGAAGAATAGGAAAAAGCCCGGAGCG
j5_00743_(CTD_downstream)_reverse	GGCTGCGCATTCCTGTGGTCTAGAGCGGCCGCCACCGCGGTGGA
nptII −136F	TAAGGATCCGGAATTGCCAGCTGGGGCGCCCTC
nptII + 3R	CCAGGATCCCGCTCAGAAGAACTCGTCAAG
SMc01336 1825F	GCAGTTCGATCACGGACTAC
SMc01336 + 285R	CGTGACCGAATAATGCAGAG
KM44	TACTTCCAATCCAATGCAATGGCAGAGAAAATGCT
KM45	TTATCCACTTCCAATGTTATTATTAGAAGAAGCCGCGG
KM85	GAAGAAGAGGTCCTCATCGA
KM91	CATATTGGATTGGAAGTACAGGTTTTC
KM83	GTCTTCCGGCTCCGGCTC
KM90	TAAATTGGAAGTGGATAACGGATCCG

#### JOE6516: Rm1021 *rne*ΔIDR

*S. meliloti* RNase EΔIDR mutants, *rne*^+^ correction, and *C. crescentus rne* IDR hybrid strains were generated via two-step allelic replacement by homologous recombination, using plasmids derived from pJQ200sk (44). pJSE3 was used to delete the region coding for the C-terminal IDR. pJSE3 was generated as follows.

First, 1,000 bp upstream and 1,000 bp downstream of the region encoding the RNase E C-terminal domain (CTD) were amplified by PCR from the Rm1021 genome with primers j5_00743_(CTD_upstream)_forward, j5_00743_(CTD_upstream)_reverse, j5_00743_(CTD_downstream)_forward and j5_00743_(CTD_downstream)_reverse, designed using the j5 DNA assembly design automation software (Table 3) (46). Next, the resulting upstream and downstream PCR fragments were gel-purified, assembled into pJQ200sk via Gibson cloning, and transformed into *E. coli* DH5α (Invitrogen). Gent^R^ colonies were then screened by PCR for both inserts, and purified plasmids were verified by Sanger sequencing (Genewiz).

JOE6526 (Rm1021 *podJ2..nptII rne*ΔIDR) was also constructed by using pJSE3 to generate the deletion in JOE6312 (Rm1021 *podJ2..nptII*), whereas JOE6550 (Rm1021 *podJ2*..Ω *rne*ΔIDR) was constructed by transducing the *podJ2*..Ω allele from JOE2612 (43) into JOE6516.

JOE6312 was generated by introducing the *nptII* gene into Rm1021, downstream of SMc02231, using the pJQ200sk-derived plasmid pJC739, which was constructed by amplifying the *nptII* gene from pBbB2k-GFP (47) with primers nptII −136F and nptII +3R (Table 3), digesting the resulting PCR product with BamHI, and inserting the fragment into the BamHI site of pJC377 (parent of pJC382) (43).

#### JOE6831: Rm1021 *rne^+^*

Plasmid pJSE4 was used to reintroduce the wild-type *rne* allele into JOE6516. pJSE4 was generated by amplifying from 1,000 bp upstream to 1,000 bp downstream of the region encoding the RNase E CTD from the Rm1021 genome with primers j5_00743_(CTD_upstream)_forward and j5_00743_(CTD_downstream)_reverse, designed using the j5 DNA assembly design automation software (Table 3) (46). Next, the resulting PCR product was gel-purified, inserted into pJQ200sk via Gibson assembly, and transformed into *E. coli* DH5α (Invitrogen). Gent^R^ colonies were then screened by PCR for the full insert, and purified plasmids were verified by Sanger sequencing (Genewiz). Then, pJSE4 was used to generate the *rne*^+^ strain via two-step allelic replacement by homologous recombination.

JOE6825 (Rm1021 *podJ2..nptII rne^+^*) and JOE6826 (Rm1021 *podJ2*..Ω *rne^+^*) were similarly constructed via two-step allelic replacement from JOE6526 or JOE6550, respectively.

#### JOE7534: Rm1021 *rne C. crescentus* IDR hybrid

Plasmid pKM40 was used to introduce the *C. crescentus rne* IDR into JOE6516. pKM40 was generated by cutting pJSE4 with MIuI and XhoI, then performing a Gibson assembly (NEB) with a *C. crescentus rne* IDR gene fragment synthesized by Twist Bioscience.

Gent^R^ colonies were screened by restriction digestion for the full insert, and purified plasmids were verified by whole-plasmid sequencing (Genewiz). Then, pKM40 was used to generate the *C. crescentus* IDR hybrid strain via two-step allelic replacement by homologous recombination, as above.

### pKM3, pKM44 and pKM45

pET His6 MBP TEV LIC cloning vector (1M) was used as the base vector to generate pKM3, pKM44, and pKM45 plasmids. The pET His6 MBP TEV LIC cloning vector (1M) was a gift from Scott Gradia (Addgene plasmid #29656; http://n2t.net/addgene:29656; RRID:Addgene_29656). To generate pKM3, the *rne* gene was amplified from *S. meliloti* Rm1021 genomic DNA using primers KM44 and KM45 (Table 3). The pET His6 MBP TEV LIC cloning vector was linearized using the SspI restriction enzyme. The amplified gene was then incorporated into the linearized vector using ligation-independent cloning and transformed into *E. coli* DH5α cells. Resulted KanR colonies were grown overnight in LB medium, and plasmids were miniprepped. Miniprepped plasmids were screened by restriction digestion for the full insert and verified by whole-plasmid sequencing (Genewiz).

pKM44 was generated by inverse PCR of pKM3 using primers KM85 and KM91 (Table 3), followed by DpnI treatment, agarose gel purification of the amplicon, ligation using T4 DNA ligase (Thermo Scientific) and transformation into *E. coli* DH5α cells. pKM45 was generated by a similar inverse PCR of pKM3 using primers KM83 and KM90 (Table 3), followed by DpnI treatment, agarose gel purification, ligation and transformation. KanR colonies resulted from tramsformation were grown overnight in LB medium, plasmids were miniprepped, screened by restriction digestion, and verified by whole-plasmid sequencing (Genewiz).

### Western blotting

For the western blot, log-phase cells were pelleted and resuspended in 250 µL of 1× Laemmli buffer for each 1.0 OD_600_ unit and boiled at 95°C for 5 min; 15 µL of samples were loaded in a 4%–20% Bio-Rad stain-free TGX precast gel alongside 1 µL of Positope Control Protein (Thermofisher R90050) and run at 150V for 60 min. Semi-dry transfer was done using the BioRad Trans-Blot Turbo transfer system and Trans-Blot Turbo midi 0.2 µm nitrocellulose transfer pack according to the manufacturer’s instructions. Blocking was done for 1 h using 15 mL of 3% BSA in TBST (20 mM Tris, pH 7.5, 150 mM NaCl, 0.1% Tween-20) at room temperature with gentle shaking. For primary antibody binding, the membrane was submerged in a 1:1,000 dilution of the anti-GFP antibody (Millipore Sigma 11814460001) in the same blocking buffer and shaken gently overnight at 4°C. After washing the membrane five times with TBST for 10 min each time, the membrane was probed with 1:20,000 goat anti-mouse IgG secondary antibody with HRP conjugate (Millipore Sigma 12-349) in blocking buffer. Secondary antibody incubation was done for 1 h with gentle shaking at room temperature. The membrane was then washed five times, 10 min each time, with 1× TBST with gentle shaking. Following the wash, the blot was revealed with SuperSignal West Pico PLUS chemiluminescent reagent (ThermoFisher 34577) for 5 min and imaged using the Bio-Rad ChemiDoc MP imaging system.

### Cell imaging and analysis

For imaging, cells were immobilized on 1.5% agarose pads made with 1x M9 salts on microscope slides, and images were collected using a Nikon Eclipse NI-E with CoolSNAP MYO-CCD camera and 100× Oil CFI Plan Fluor (Nikon) objective, driven by NIS-Elements software. The Chroma 96363 filter set was used for YFP. For time-lapse experiments, images were taken at 1 min intervals with manual focus over a maximum time period of 30 min, and approximately 5–8 fusion events were observed per movie.

Cell image analysis was performed using MicrobeJ (48). For automated foci detection, the maxima foci function of MicrobeJ was used. The tolerance and Z-score settings were manually adjusted to identify and outline foci on a test image and then to remove aberrant foci with an areaof <0.01 µm^2^ and a lengthof >1 µm. The segmentation option was also used to split adjoined foci. For a given set of images, the same tolerance/Z-score parameters were applied to quantify the number of foci per cell with a minimum of 50 cells.

### Protein purification and *in vitro* assays

BL21(DE3) cells transformed with the pET-MBP vector bearing the 6×-His-MBP-RNase E, -RNase EΔIDR, or -RNase E IDR only gene under the control of the IPTG-inducible promoter (pKM3, pKM44 or pKM45) were grown in 2 L of 2× LB medium until OD600 reached 0.6–0.7. The cells were then induced with 0.5 mM IPTG at 30°C for 3.5 h. The induced cells were centrifuged at 8,000 × *g* for 10 min, and the pellet was resuspended in 30 mL lysis buffer (20 mM Tris, pH 7.4, 0.5 M NaCl, 10% glycerol, 20 mM imidazole, supplemented with 40 µg/ml DNase I, 1 mM PMSF, and three protein cocktail inhibitor tablets). The resuspended cells were lysed on ice using a sonicator at 50% intensity with 7 s pulse-on and 10 s pulse-off cycles for 4 min. The lysate was centrifuged at 15,000 × *g* for 45 min, and the supernatant was applied to 10 mL pre-equilibrated nickel-NTA resin in a gravity flow column. The protein-bound column was then washed with 100 mL lysis buffer, 100 mL high salt buffer (20 mM Tris, pH 7.4, 1 M NaCl, 10% glycerol, 10 mM imidazole), 100 mL wash buffer (20 mM Tris, pH 7.4, 150 mM NaCl, 10% glycerol, 10 mM imidazole), and eluted in elution buffer (20 mM Tris, pH 7.4, 150 mM NaCl, 5% glycerol, 250 mM imidazole) as 5 mL fractions. The protein-containing fractions were pooled and concentrated to approximately 10 mg/mL. The concentrated protein was passed through a Superdex 200 Increase 10/300 Gl size exclusion column in SEC buffer (20 mM Tris, pH 7.4, 250 mM NaCl, 2% glycerol, 1 mM DTT). The protein was then concentrated to approximately 10 mg/mL and stored at −80°C.

When necessary, the 6×-His-MBP tag was removed by incubating 6×-His-MBP-RNase E protein with TEV protease in SEC buffer at a molar ratio of 20:1, overnight at 4°C. The protein sample was then passed through Superdex 200 Increase 10/300 Gl size exclusion column to separate RNase E from the 6×-His-MBP. RNase E was then concentrated to approximately 10 mg/mL and stored in −80°C.

To perform the *in vitro* phase separation assays, the 6×-His-MBP tag was removed, and 6 µM of the protein was incubated with or without 25 ng/µL *E. coli* total RNA in a 20 mM Tris, pH 7.4, 100 mM NaCl, 1 mM DTT buffer in a total reaction volume of 10 µL for 30 min at room temperature. The entire 10 µL was spotted on a slide and covered with a coverslip before imaging with Nikon Eclipse NI-E with CoolSNAP MYO-CCD camera and 100× Oil CFI Plan Fluor (Nikon) objective under phase contrast, with 30 ms exposure.

To test the RNA decay activity of the purified proteins, 0.1 µM *E. coli* 9S RNA was incubated with 0.2 µM of the protein in 20 mM Tris, pH 7.4, 150 mM NaCl, 2% glycerol, 1 mM DTT, and 5 mM MgCl_2_ buffer in a total reaction volume of 30 µL at 28°C for 5 and 15 min. At the end of each time point, 10 µL of the reaction volume was mixed with 15 µL of stop buffer (95% formamide, 50 mM ethylenediamine tetra-acetic acid [EDTA], 0.1% SDS, 0.025% bromophenol blue, and 0.025% xylene cyanol). The samples were heated at 90°C for 3 min, and 10 µL of the sample was loaded and resolved on 7% urea-acrylamide gel, stained with 1× SYBR Gold nucleic acid stain, and scanned using an iBright imaging system.

### RNA decay rates by RNA-seq

Cells were grown in TY media overnight. The next day, mid-log phase cultures (OD600 0.3–0.5) were treated with 10 mg/mL rifampicin to achieve a final concentration of 200 µg/mL. Before adding rifampicin, 1 mL of culture (0 minutes) was removed and mixed with 2 mL of RNAprotect Bacterial Reagent (QIAGEN), vortexed, and incubated at room temperature for 5 min. This step was repeated at 1, 3, and 9 min after rifampicin was added. The cells were pelleted at 20,000 × *g* for 1 min and resuspended in 1 mL of 65°C TRIzol Reagent (Ambion) and incubated at 65°C for 10 min in a heat block. 200 µL of chloroform was added to the samples, and the tubes were incubated at room temperature for 5 min before spinning at 20,000 × *g* for 10 min. RNA samples were chloroform extracted once and precipitated using isopropanol (1× volume isopropanol, 0.1× volume 5 M sodium acetate, pH 5.2) overnight at −80°C. The RNA samples were spun at 20,000 × *g* at 4°C for 1 h; the pellets were washed with 80% ethanol for 10 min, air dried, and resuspended in 10 mM Tris-HCl (pH 7.0). The RNA-Seq libraries were made using 5 µg of total RNA samples, and library construction was performed according to published protocol (49). To measure RNA-decay rates, the fraction of mRNA remaining was calculated as the RPKM of each time point divided by the RPKM measured in the untreated 0 min sample (Data set S1). For bulk mRNA half-life calculations of RNA-seq data, the fraction of all mRNA reads was compared with the total fraction of reads, which includes a majority of stable tRNA reads. Half-lives of individual mRNAs were calculated using the Rifcorrect software package (Data set S2).

### Symbiosis assays with *M. truncatula*

*M. truncatula* seedlings were grown in nitrogen-free media in 18 × 150 mm test tubes and inoculated with Rm1021 or derivatives as previously described (41). Root nodules were counted every 7 days for 42 days, starting at 14 days post-inoculation (Data set S3). Plants and nodules were photographed at the end of 42 days; nodule images were captured using an Olympus SZX12 Stereozoom dissecting microscope, at 10× magnification, with a QImaging MicroPublisher 5.0 camera and associated QCapture software. To determine the chlorophyll content, foliage was harvested from each plant 42 days post-inoculation, macerated in 0.2 mL water, and boiled for 5 min; 0.8 mL acetone was added to a final concentration of 80%, and the mixtures were incubated at room temperature overnight. The crude extracts were clarified by centrifugation for 5 min at 15,000 × *g*, and the absorbance of the supernatant was measured at 645 and 663 nm. Arnon’s equations were used to estimate chlorophyll concentrations (50, 51).

Competitive colonization assays were also performed as previously described (41). Briefly, equal volumes of two cell suspensions (*S. meliloti* Rm1021 and its derivatives) were mixed for inoculating *M. truncatula* seedlings, at 10^6^–10^7^ cells per plant. Strains were marked with resistance to spectinomycin or neomycin for phenotypic identification and verification of colony-forming units (CFUs) in each inoculating mixture. Bacteria were recovered from individual nodules 28 days post-inoculation using appropriate selective media to determine the dominant strain in each nodule (>60 nodules per trial, three or more trials per competition group) (Data set S4). Nodules were individually surface sterilized with 10% bleach, washed with sterile water twice, and crushed with sterile wooden sticks in tubes containing 50 µL PYE. Each extraction was serially diluted and plated on two or more PYE media (containing neomycin, spectinomycin, or streptomycin). For one of the RNase E vs. RNase EΔIDR competitions (JOE2612 vs. JOE6550), both strains carried the spectinomycin resistance marker. In that competition, the genotype of individual isolates from the inoculating mixture and the nodule extracts was determined by PCR using the primers SMc01336 1825F and SMc01336 + 285R (Table 3). Wild-type *rne* yielded a 1,235-bp product, whereas the ΔIDR mutation yielded a 458-bp product.

All the information on statistical analysis can be found in Data set S5.

## Data Availability

Raw sequencing data are available in the NCBI GEO database with accession number GSE251703.

## SUPPLEMENTAL MATERIAL

Data set S1 mbio.02490-25-s0001.xlsx Rif-seq RPKM data.

Data set S2 mbio.02490-25-s0002.xlsx mRNA half-life calculations.

Data set S3 mbio.02490-25-s0003.xlsx Root nodule counts.

Data set S4 mbio.02490-25-s0004.xlsx Symbiosis competition data.

Data set S5 mbio.02490-25-s0005.xlsx Statistical analysis.

Supplemental material mbio.02490-25-s0006.docx Supplemental figures and legends for supplemental data sets.

ASM does not own the copyrights to Supplemental Material that may be linked to, or accessed through, an article. The authors have granted ASM a non-exclusive, world-wide license to publish the Supplemental Material files. Please contact the corresponding author directly for reuse.
