# Supplementary material for: Sinorhizobium meliloti BR-bodies promote fitness during host colonization
Source: mBio. 2025 Oct 31;16(12):e02490-25. doi: 10.1128/mbio.02490-25 (PMC12691583; doi:10.1128/mbio.02490-25)
Supplement: Supplemental material — Supplemental figures and legends for supplemental data sets. [file mbio.02490-25-s0006.docx]

**Supplementary Figures**


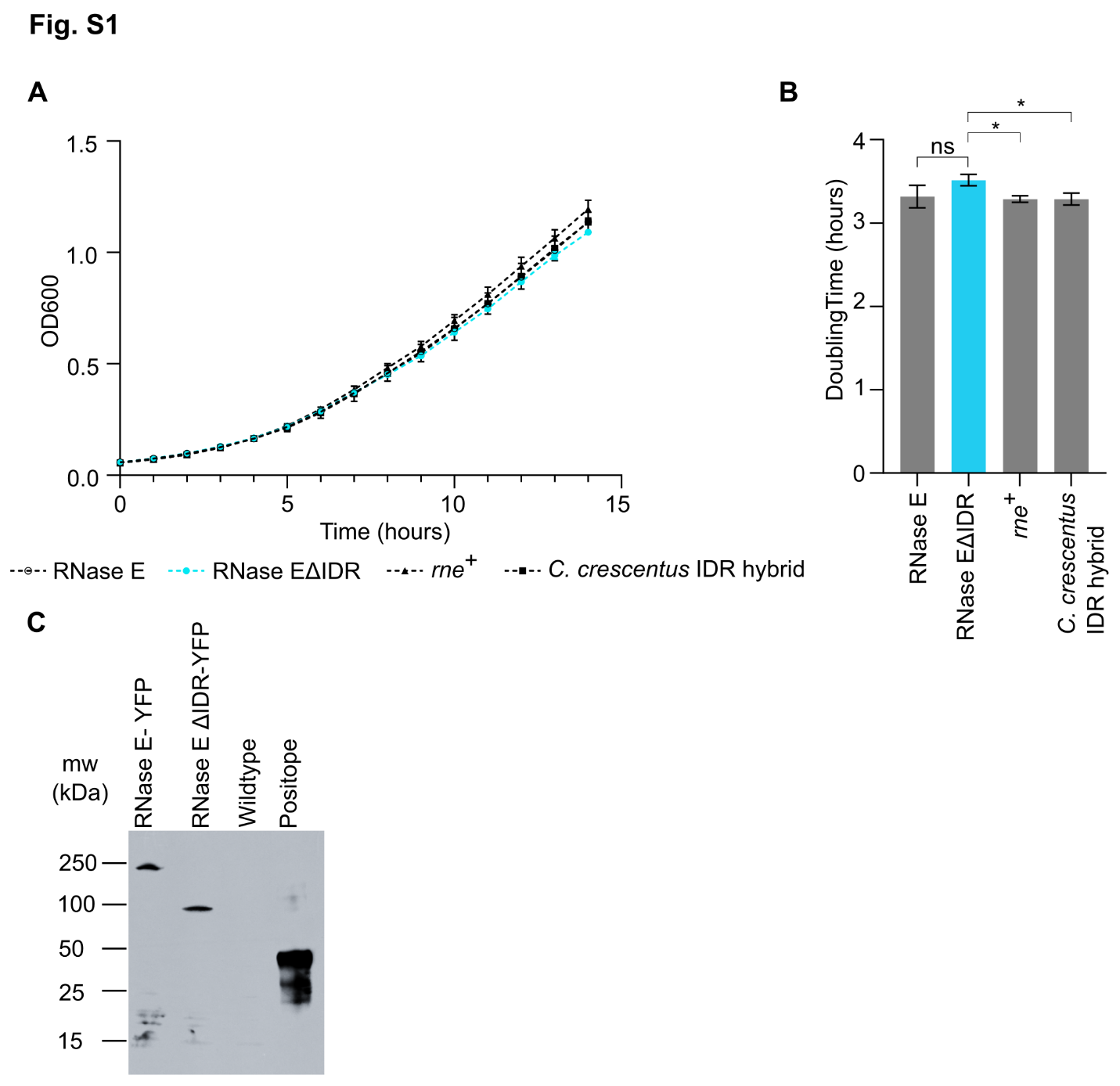


**Figure S1: Growth and expression analysis of *S. meliloti* RNase E strains. A.)** Growth curves for wild-type *S. meliloti* (RNase E), RNase EΔIDR, *rne*^+^ and *C. crescentus* IDR hybrid strains in TY media. The growth rates of 25 mL cultures were determined by measuring the optical density at 600 nm (OD600) every hour, starting at 0.05 for 14 hours, using the NanoDrop™One^C^ spectrophotometer. Error bars indicate the standard deviations from three replicates. **B.)** Doubling times for *S. meliloti* strains in TY media. Exponential curve fits were performed in mid-log phase (OD600 0.3-0.6) to calculate the average doubling times. Error bars represent standard deviations from three replicate growth curves. Two-tailed t-test with unequal variance was used to determine the p values (ns: not significant, *: p<0.05). **C.)** Western blot showing the expression levels of full-length RNase E- and RNase EΔIDR-YFP fusions in mid-log phase. Wild-type *S. meliloti* cells were used as negative control (no YFP), while the Positope (Thermo Fisher) protein was used as a positive control for primary antibody binding.


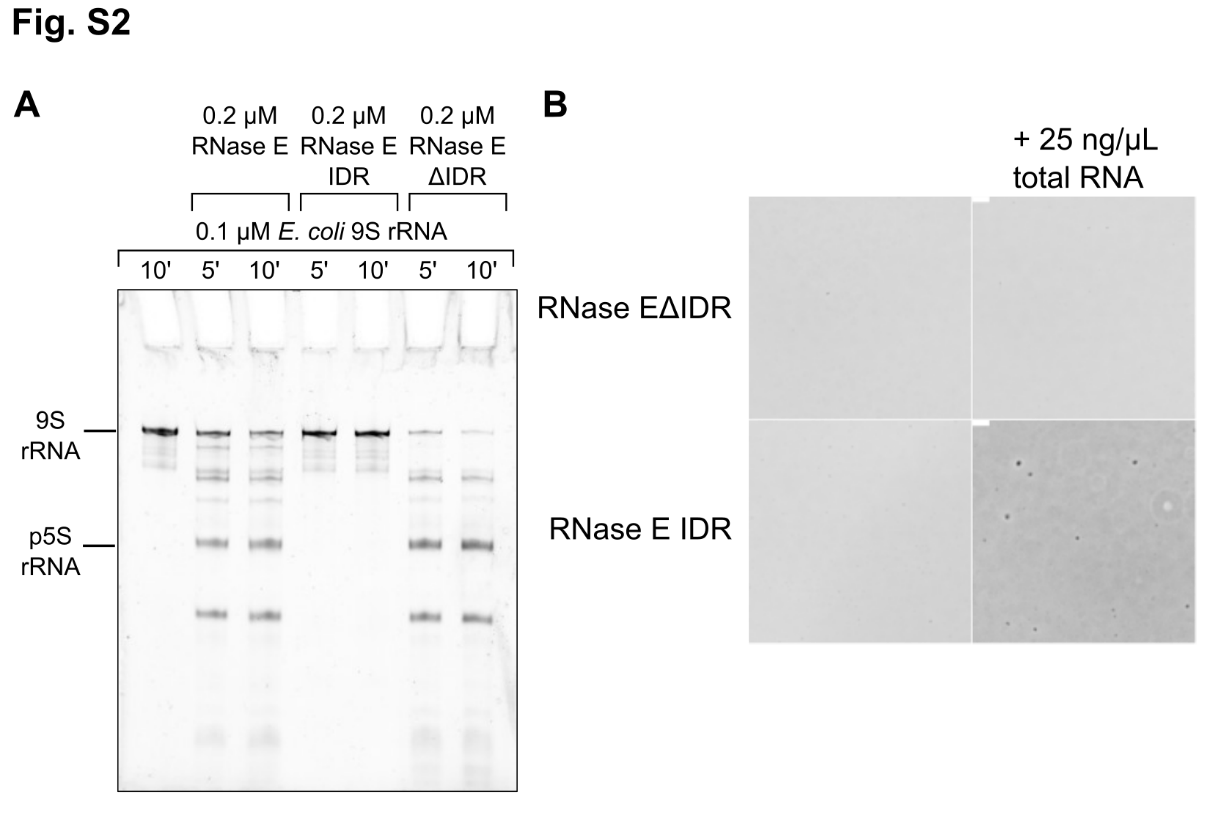


**Figure S2: 9S rRNA processing activity and phase separation of purified *S. meliloti* RNase E variants. A.)** Processing of *E. coli* 9S rRNA to 5S rRNA by purified *S. meliloti* full-length RNase E, RNase EΔIDR and RNase E IDR with the MBP tag. 0.1 μM *E. coli* 9S RNA was incubated with 0.2 μM of purified *S. meliloti* RNase E variants for 5 and 10 minutes. 9S RNA without any RNase E was incubated in the reaction buffer for 10 minutes as a negative control. The products were resolved on 7% acrylamide/bisacrylamide-urea gel. Full-length RNase E and RNase EΔIDR showed rRNA processing activity, while RNase E IDR did not show enzymatic activity. **B.)** *In vitro* phase separation of purified RNase EΔIDR and RNase E IDR. RNase EΔIDR does not undergo phase separation either in the presence or absence of RNA, while RNase E IDR alone undergoes phase separation in the presence of RNA. The white scale bar is 5 μm.


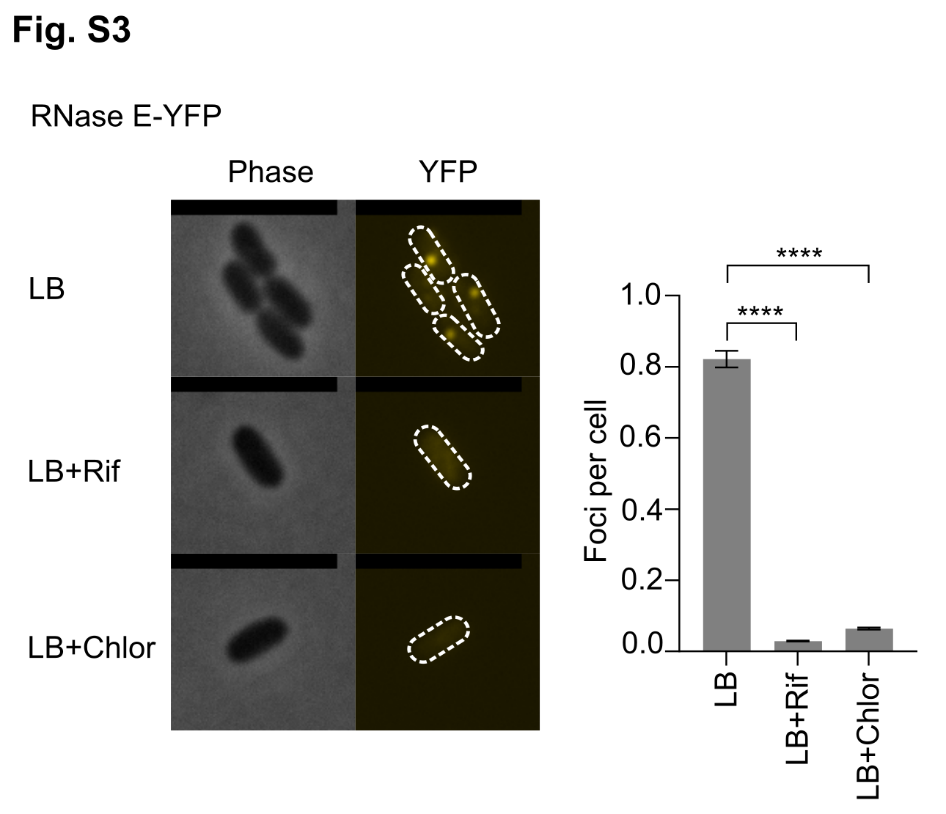


**Figure S3: BR-body formation in *S. meliloti* is RNA-dependent.** Cells expressing RNase E-YFP and grown in LB were treated with 100 µg/mL of rifampicin (Rif) for 30 minutes to deplete mRNA or 200 µg/mL of chloramphenicol (Chlor) for 30 minutes to arrest translation and accumulate mRNA in polysomes, prior to examination by microscopy and detection of fluorescence foci. Foci quantitation was performed using microbeJ to compare treated and untreated cells. 1240 cells were used for untreated cells, 482 cells were used for rifampicin-treated cells, and 467 cells were used for chloramphenicol-treated cells. p-values were calculated with t-test (two-tailed, unequal variance, ****: p < 0.0001). Error bars represent standard errors. Black scale bar is 5 μm.


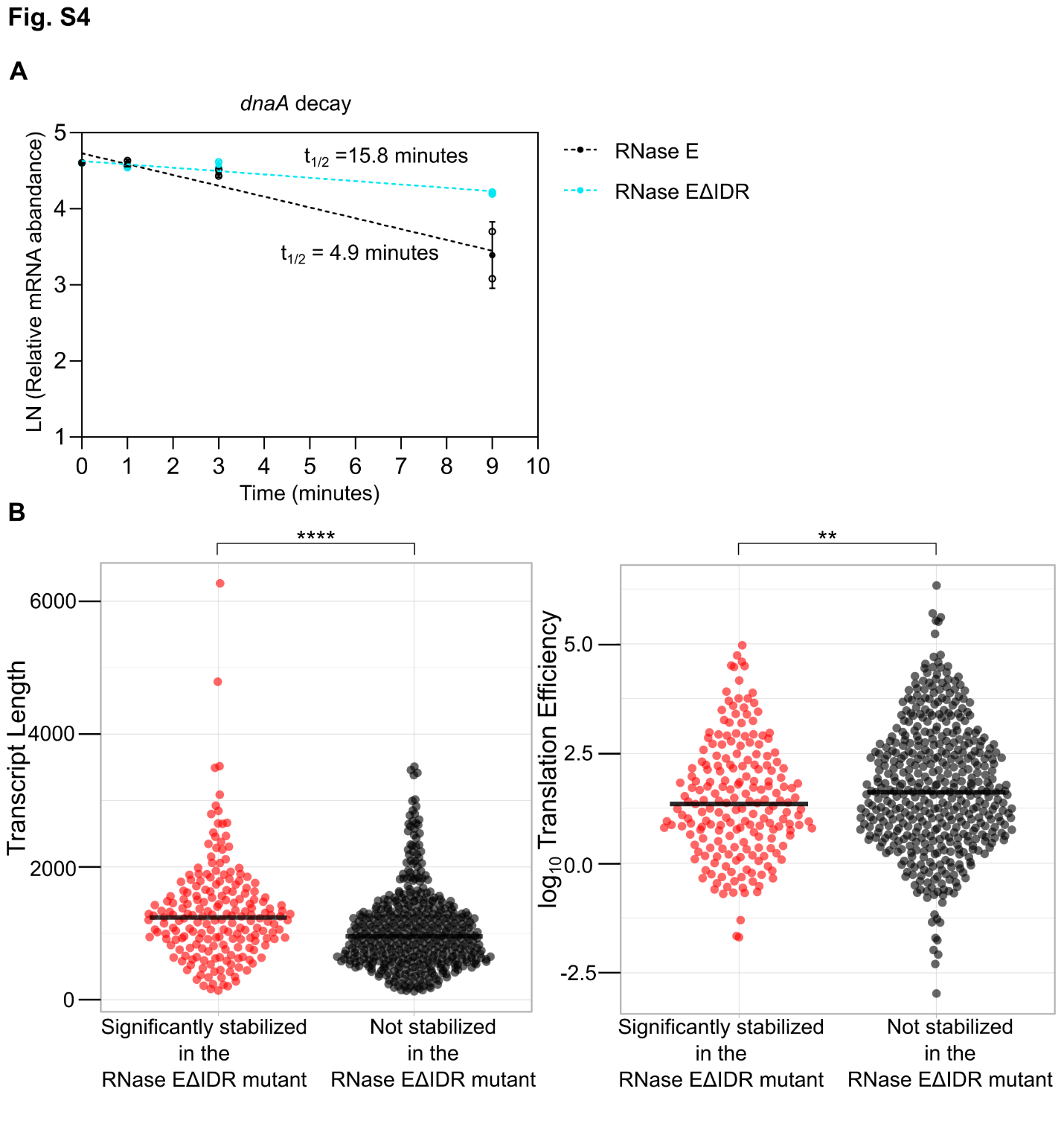


**Figure S4: *dnaA* transcript stabilization and transcriptome-wide features of mRNAs with altered half-lives in the *S. meliloti* RNase EΔIDR mutant. A.)** Decay curves of *dnaA* transcript in RNase E and RNase EΔIDR strains. Log transformed RPKM remaining at each time point relative to the 0’ time point is plotted against time, showing that the *dnaA* transcript is stabilized in the RNase EΔIDR strain. Error bars represent standard deviations from two replicate measurements. **B.)** Comparison of transcript lengths and translation efficiencies for transcripts with and without significant stabilization in the *S. meliloti* RNase EΔIDR mutant. Transcripts with significant stabilization (p < 0.05) in the RNase EΔIDR mutant, as identified by Rif-seq, were significantly longer (p < 0.0001) and had significantly lower translation efficiencies (p < 0.01) compared to transcripts that were not significantly stabilized (p > 0.05). Translation efficiency and mRNA length values were obtained from a published ribosome profiling dataset. Statistical significance was assessed using one-tailed t-test with unequal variance (**: p < 0.01; ****: p < 0.0001).


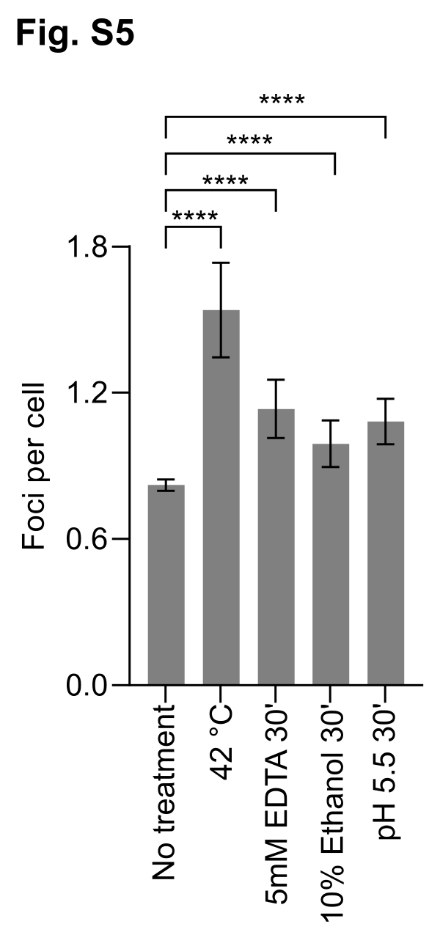


**Figure S5: *S. meliloti* BR-bodies are strongly induced under stress.** RNase E-YFP cells were grown in TY medium and then treated with the indicated stresses for 30 minutes before being placed on TY 1.5% agarose pads for imaging. >100 cells were analyzed for each condition, and the average foci per cell was calculated using microbeJ. The error bars represent standard errors. p-values were calculated with t-test (one-tailed t-test, unequal variance, ****: p < 0.0001).


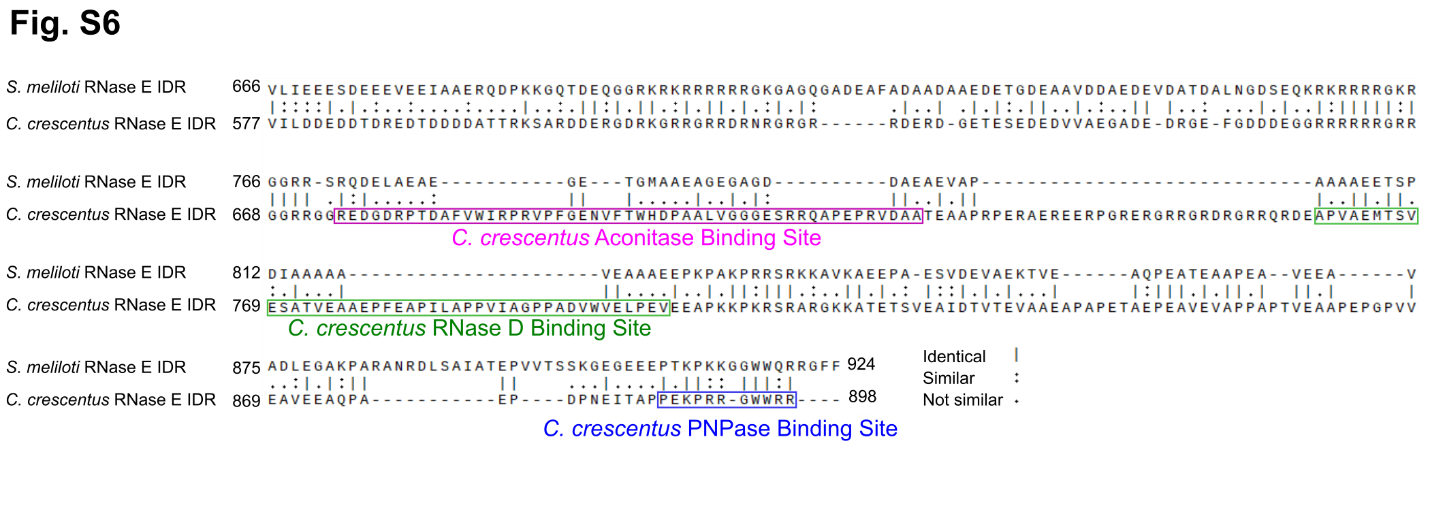
**Figure S6: *S. meliloti* RNase E IDR and RNA degradosome binding sites are highly divergent from those in *C. crescentus*.** The *S. meliloti* RNase E IDR shares only 28.86% overall sequence identity with the *C. crescentus* RNase E IDR (Needleman-Wunsch global alignment, alignment matrix: BLOSUM62, gap open penalty:10.0, gap extend penalty: 1.0). Known *C. crescentus* degradosome binding sites within the IDR also show low sequence identity when aligned to the *S. meliloti* IDR: aconitase binding site (21.05%), RNase D binding site (36.11%), and PNPase binding site (58.33%) (Smith-Waterman local alignment, alignment matrix: BLOSUM62, gap open penalty:10.0, gap extend penalty: 1.0).


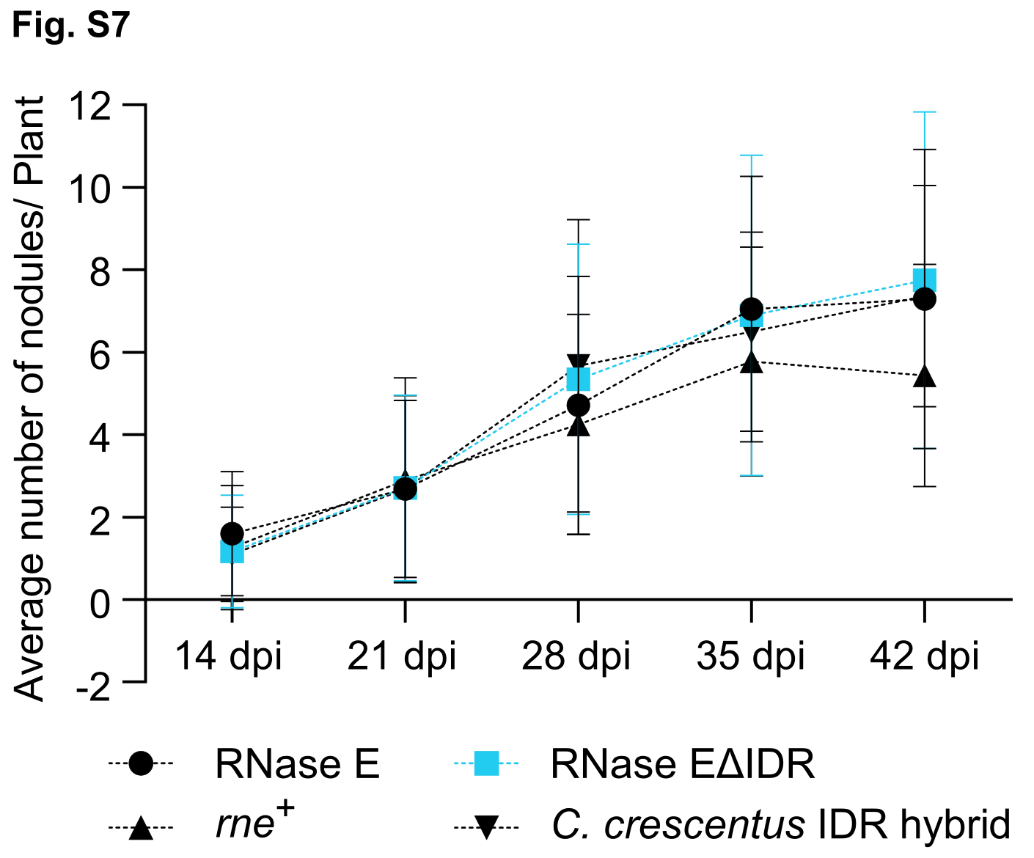


**Figure S7: RNase EΔIDR allows root nodule formation but impairs development of pink nodules in the host plant *M. truncatula*.** The number of root nodules per plant and their colors were recorded every seven days, starting at 14 days post-inoculation (dpi) (Table S3). The average number of nodules per plant among plants inoculated with RNase E, RNase E ΔIDR*, rne+ or C. crescentus* IDR hybrid were compared using a two-tailed t-test with unequal variance and the resulting p-values are listed in Table S3. Error bars represent standard deviations. Uninoculated plants did not develop any nodules.

**Supplementary Dataset Legends**

**Dataset S1: Rif-seq RPKM data.** RPKM data for two replicates of RNase E and RNase E ΔIDR strains at 0,1,3 and 9 minute timepoints after adding Rifampicin.

**Dataset S2: mRNA half-life calculations.** Half-lives of individual mRNAs calculated using the Rifcorrect software package.

**Dataset S3: Root nodule counts.** The number of root nodules per *M. truncatula* plant inoculated with *S. meliloti* RNase E, RNase E ΔIDR*, rne+ or C. crescentus* IDR hybrid strains.

**Dataset S4: Symbiosis competition data.** Symbiosis competition between RNase E vs. RNase E (R), or RNase E vs. RNase EΔIDR in *M. truncatula*.

**Dataset S5: Statistical analysis.** Statistical analysis used for all the experiments throughout this study.
